# Supplementary material for: Control of Golgi- V-ATPase through Sac1-dependent co-regulation of PI(4)P and cholesterol
Source: Nat Commun. 2025 Aug 21;16:7808. doi: 10.1038/s41467-025-63125-7 (PMC12371084; doi:10.1038/s41467-025-63125-7)
Supplement: Supplementary file 1 — Supplementary Information [file 41467_2025_63125_MOESM1_ESM.pdf]

Control of Golgi- V-ATPase through Sac1-dependent co-regulation of PI(4)P and cholesterol

Xin Zhou[1,2*], Miesje M. van der Stoel[1,2*], Shreyas Kaptan[3], Haoran Li[1,2], Shiqian Li[1,2], Maarit Hölttä[1,2], Helena Vihinen[4], Eija Jokitalo[4], Christoph Thiele[5], Olli Pietiläinen[6], Shin Morioka[7], Junko Sasaki[7], Takehiko Sasaki[7], Ilpo Vattulainen[3] and Elina Ikonen[1,2]

[1]Stem Cells and Metabolism Research Program and Dept. of Anatomy, Faculty of Medicine, University of Helsinki, Helsinki, Finland; [2]Minerva Foundation Institute for Medical Research, Helsinki, Finland; [3]Department of Physics, University of Helsinki, Helsinki, Finland; [4]Institute of Biotechnology, University of Helsinki, Helsinki, Finland; [5]Life and Medical Science Institute, University of Bonn, Bonn, Germany; [6]Neuroscience Center, Helsinki Inst. of Life Science, University of Helsinki, Helsinki, Finland; [7]Medical Research Institute, Tokyo Medical and Dental University, Tokyo, Japan.

*Equal contribution

Supplementary Information:

Supplementary Figure 1
Supplementary Figure 2
Supplementary Figure 3
Supplementary Figure 4
Supplementary Figure 5
Supplementary Figure 6
Supplementary Figure 7
Supplementary Figure 8

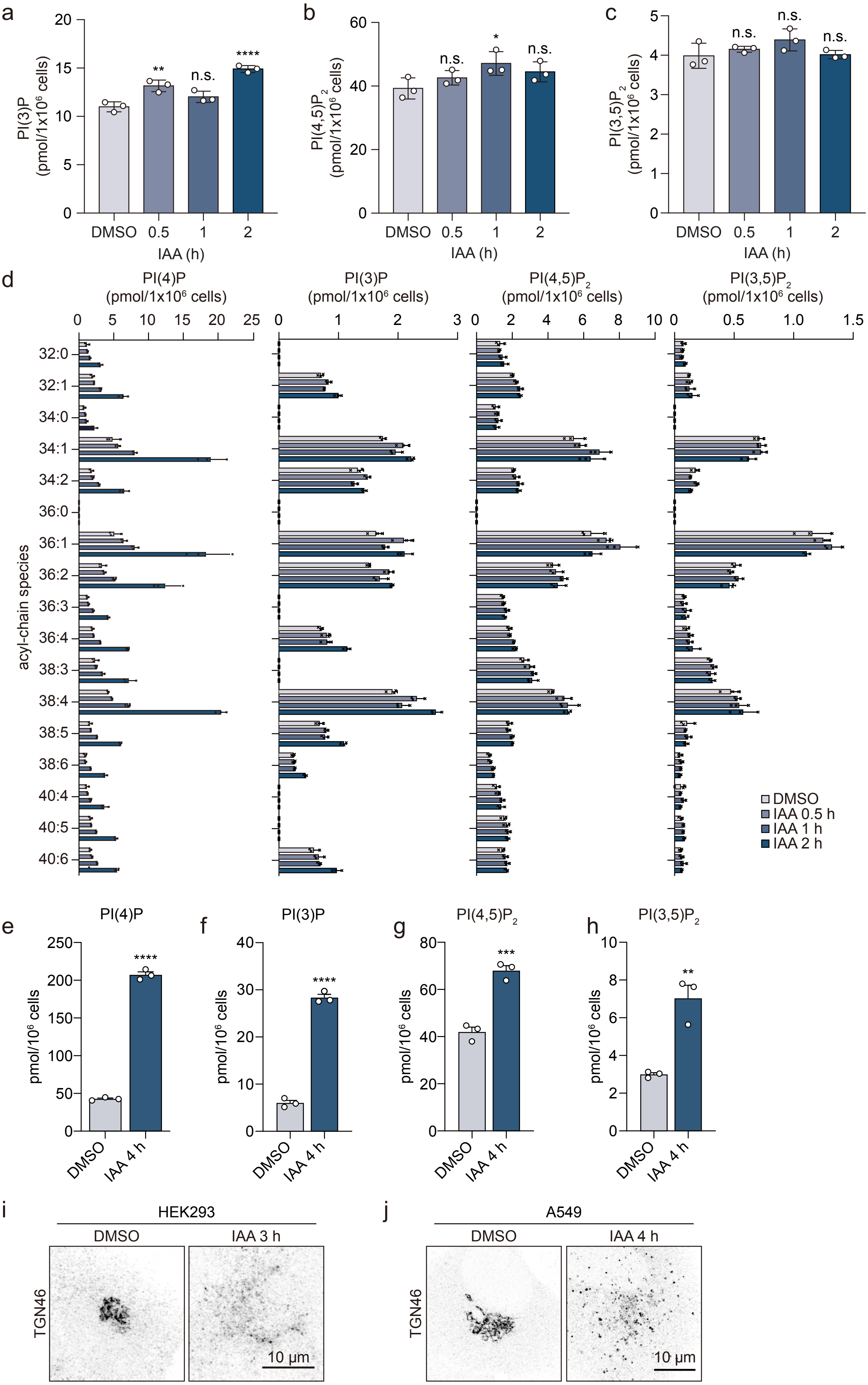

a
b
c
d
e
f
g
h
i
j
PI(3)P (pmol/1x10⁶ cells)
PI(4,5)P2 (pmol/1x10⁶ cells)
PI(3,5)P2 (pmol/1x10⁶ cells)
DMSO 0.5 1 2
IAA (h)
** n.s. **** n.s. * n.s. n.s. n.s. n.s.
PI(4)P (pmol/1x10⁶ cells)
acyl-chain species
32:0
32:1
34:0
34:1
34:2
36:0
36:1
36:2
36:3
36:4
38:3
38:4
38:5
38:6
40:4
40:5
40:6
DMSO
IAA 0.5 h
IAA 1 h
IAA 2 h
PI(4)P
PI(3)P
PI(4,5)P2
PI(3,5)P2
pmol/10⁶ cells
DMSO
IAA 4 h
**** **** *** **
HEK293
A549
DMSO
IAA 3 h
IAA 4 h
TGN46
10 µm

Supplementary Figure 1.

a-c) Graphs showing the total PI(3)P (**a**) PI(4,5)P_2 (**b**) and PI(3,5)P_2 (**c**) levels (pmol/x10^6 cells) ±S.E.M. of Sac1-degron A431 cells treated with DMSO for 2 h or IAA for 0.5, 1 or 2 h as measured by PRMC-MS. n = 3 biological replicates. One-way ANOVA with Dunnett's multiple comparisons test. PI(3)P: ** p=0.0026, n.s. p=0.0998, **** p<0.0001. PI(4,5)P2: n.s. p=0.4700, * p=0.0395, n.s. p=0.1783. PI(3,5)P2: n.s. p=0.6975, p=0.1324, p=0.9974.

d) Bar graphs depicting the average PI(4)P, PI(3)P, PI(4,5)P_2 and PI(3,5)P_2 levels (pmol/x10^6 cells) ±S.E.M. per molecular species of Sac1-degron A431 cells treated with DMSO for 2 h or IAA for 0.5, 1 or 2 h as measured by PRMC-MS.

e-h) Bar graphs depicting the average levels of PI(4)P (**e**), PI(3)P (**f**), PI(4,5)P_2 (**g**), PI(3,5)P_2 (**h**) (pmol/x10^6 cells) ±S.E.M. of Sac1-degron A431 cells treated with DMSO or IAA for 4 h as measured by PRMC-MS. n = 3 biological replicates. Two-tailed unpaired Student's t-test. PI(4)P **** p<0.0001, PI(3)P: **** p<0.0001, PI(4,5)P2: *** p=0.0009, ** p=0.0046.

i, j) Representative confocal images of Sac1-degron HEK293A cells (**i**) and A549 cells (**j**) treated for, respectively, 3 or 4 h with DMSO or IAA and stained for TGN46.

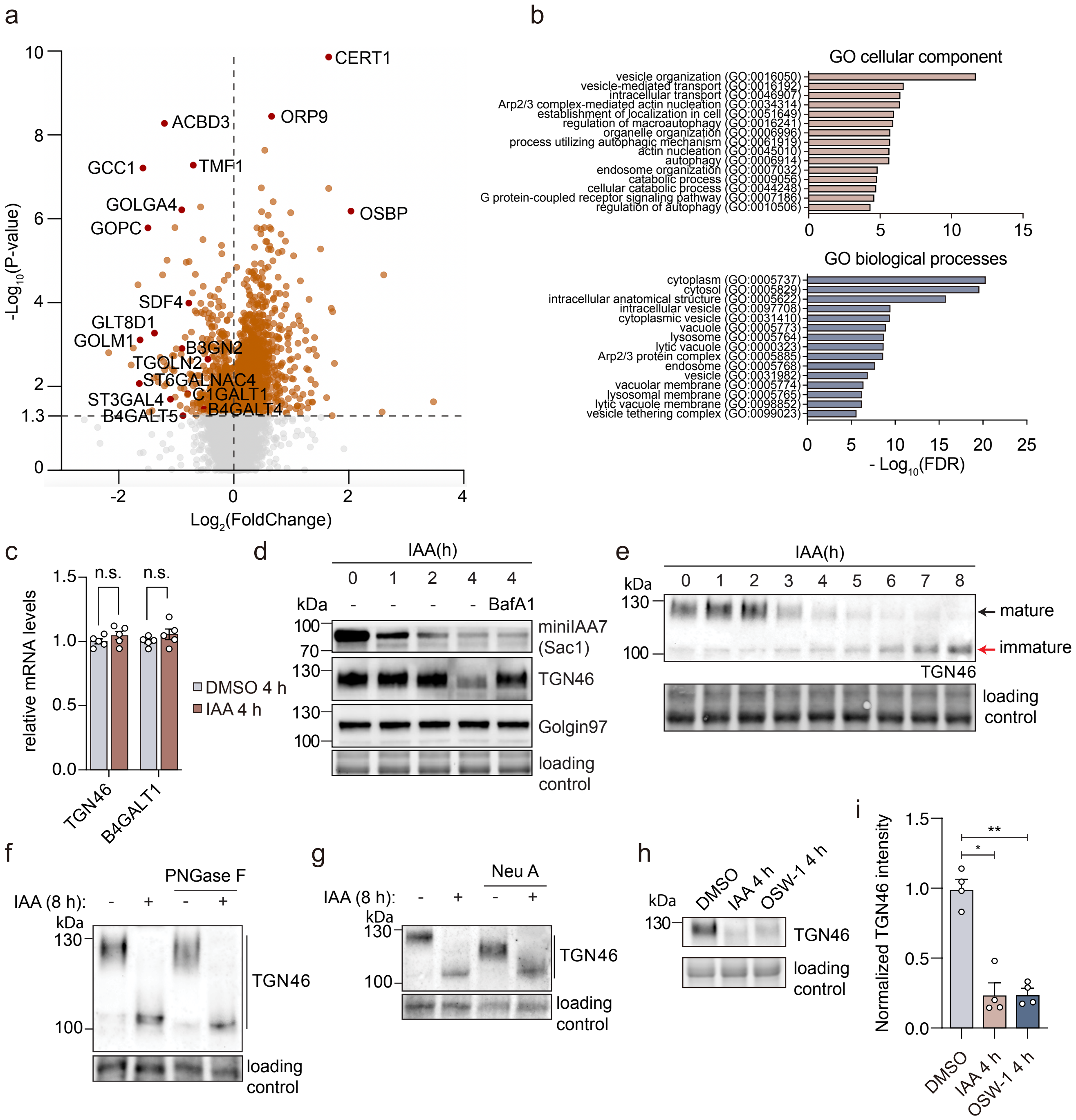
a
CERT1
ORP9
ACBD3
GCC1
TMF1
GOLGA4
OSBP
GOPC
SDF4
GLT8D1
GOLM1
B3GN2
TGOLN2
ST6GALNAC4
C1GALT1
ST3GAL4
B4GALT4
B4GALT5
-Log10(P-value)
Log2(FoldChange)
b
GO cellular component
vesicle organization (GO:0016050)
vesicle-mediated transport (GO:0016192)
intracellular transport (GO:0046907)
Arp2/3 complex-mediated actin nucleation (GO:0034314)
establishment of localization in cell (GO:0051649)
regulation of macroautophagy (GO:0016241)
organelle organization (GO:0006996)
process utilizing autophagic mechanism (GO:0061919)
actin nucleation (GO:0045010)
autophagy (GO:0006914)
endosome organization (GO:0007032)
catabolic process (GO:0009056)
cellular catabolic process (GO:0044248)
G protein-coupled receptor signaling pathway (GO:0007186)
regulation of autophagy (GO:0010506)
GO biological processes
cytoplasm (GO:0005737)
cytosol (GO:0005829)
intracellular anatomical structure (GO:0005622)
intracellular vesicle (GO:0097708)
cytoplasmic vesicle (GO:0031410)
vacuole (GO:0005773)
lysosome (GO:0005764)
lytic vacuole (GO:0000323)
Arp2/3 protein complex (GO:0005885)
endosome (GO:0005768)
vesicle (GO:0031982)
vacuolar membrane (GO:0005774)
lysosomal membrane (GO:0005765)
lytic vacuole membrane (GO:0098852)
vesicle tethering complex (GO:0099023)
- Log10(FDR)
c
n.s.
relative mRNA levels
DMSO 4 h
IAA 4 h
TGN46
B4GALT1
d
IAA(h)
0 1 2 4 4
kDa
BafA1
miniIAA7 (Sac1)
TGN46
Golgin97
loading control
e
IAA(h)
0 1 2 3 4 5 6 7 8
mature
immature
TGN46
loading control
f
PNGase F
IAA (8 h):
TGN46
loading control
g
Neu A
IAA (8 h):
TGN46
loading control
h
DMSO
IAA 4 h
OSW-1 4 h
TGN46
loading control
i
Normalized TGN46 intensity
DMSO
IAA 4 h
OSW-1 4 h

Supplementary Figure 2.

a) Volcano plot showing the up and downregulated proteins by comparing the proteome profile of 3 h IAA treated Sac1-degron A431 cells to DMSO control.

b) Top terms in gene ontology enrichment analysis of the upregulated proteins in Sac1-degron A431 cells treated with IAA for 3 h compared to DMSO control (-Log_{10} False Discovery Rate (FDR) with a fold change threshold of ≥ 1.30 or ≤ 0.77 and an adjusted p-value of ≤ 0.05).

c) Relative mRNA levels of TGN46 and B4GALT1 of Sac1-degron A431 cells treated for 4 h with DMSO or IAA. n = 5 biological replicates. Two-way ANOVA with Šídák multiple comparisons test. n.s. TGN46: p=0.3426, B4GALT1: p=0.2213.

d) Western blot analysis of Sac1-degron A431 cells treated with IAA for 0 to 4 h with or without bafilomycin A (BafA1). Blotted for miniIAA7 (Sac1), TGN46 and Golgin-97.

e) Western blot analysis of Sac1-degron A431 cells treated for 0 to 8 h with IAA and blotted for TGN46. Black arrow indicates mature TGN46, and red arrow indicates the immature form of TGN46.

f) Western blot analysis of Sac1-degron A431 cells treated for 8 h with DMSO or IAA and with or without PNGaseF digestion. Blotted for TGN46.

g) Western blot analysis of Sac1-degron A431 cells treated for 8 h with DMSO or IAA and with or without α2-3,6,8,9 Neuraminidase A digestion. Blotted for TGN46.

h) Western blot analysis of Sac1-degron A431 cells treated for 4 h with DMSO, IAA or 20 nM OSW-1. Blotted for TGN46.

i) Bar graph showing the average normalized intensity of TGN46 in **(h)**. n = 4 independent experiments. One-way ANOVA with Dunnet’s test for multiple comparisons. * p=0.0212, ** p=0.0035.

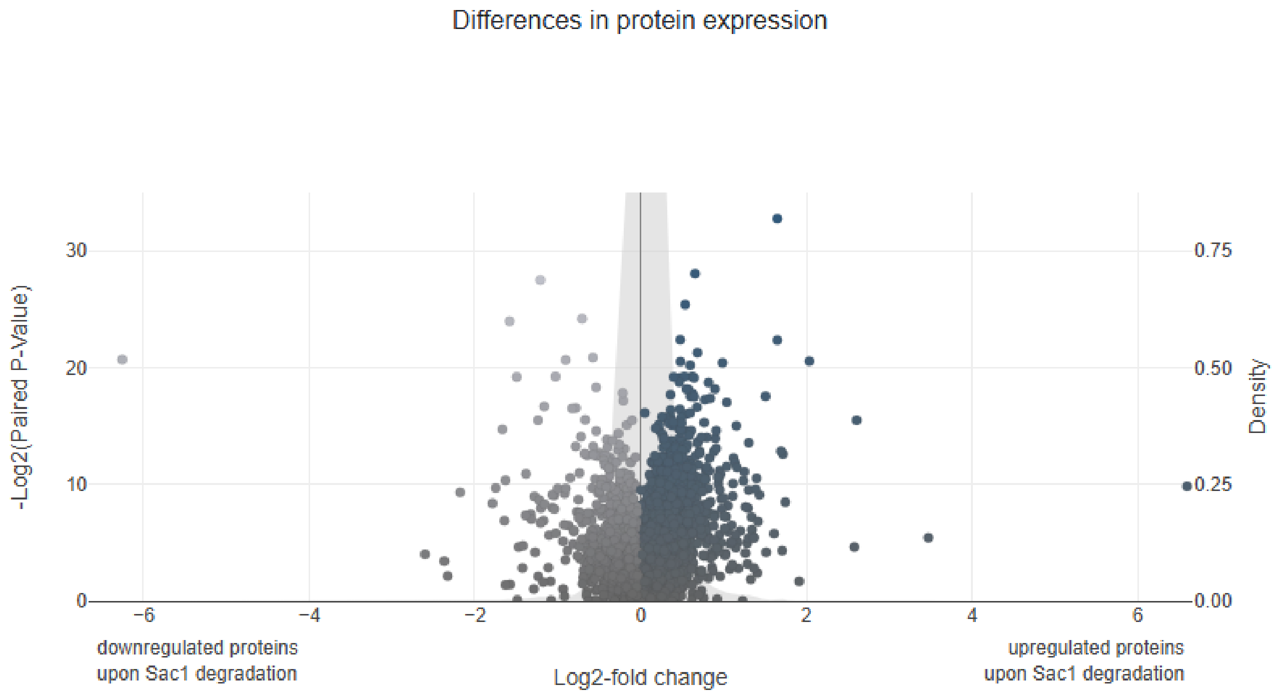

Supplementary Figure 3.

Interactive volcano plot of the proteomics analysis of Sac1-degron A431 cells: Sac1_Supplementary_Info_1_CVX-vs-DMSO_multiSelect_mod.html. The volcano plot depicts the up- and downregulated proteins by comparing the proteome profile of 3 h IAA treated Sac1-degron A431 cells to DMSO control. The data points show the differences in protein abundance in Sac1 degraded cells. Holding the cursor over a data point shows the protein name, UNIPROT ID, and a description of the protein.

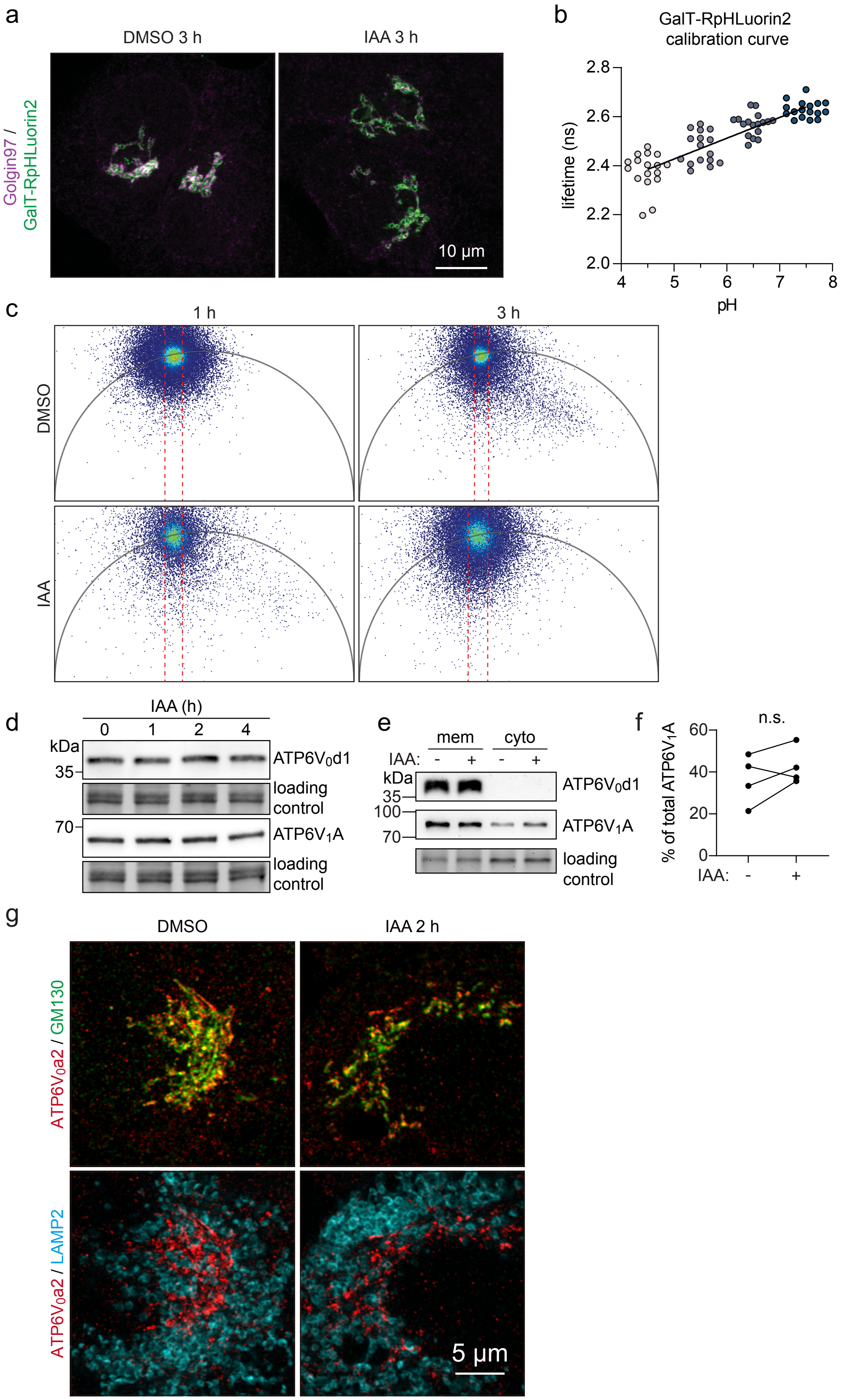

a
DMSO 3 h
IAA 3 h
Golgin97 / GalT-RpHLuorin2
10 µm
b
GalT-RpHLuorin2 calibration curve
lifetime (ns)
2.8
2.6
2.4
2.2
2.0
4
5
6
7
8
pH
c
1 h
3 h
DMSO
IAA
d
IAA (h)
0
1
2
4
kDa
35
70
ATP6V0d1
loading control
ATP6V1A
loading control
e
mem
cyto
IAA:
-
+
kDa
35
100
70
ATP6V0d1
ATP6V1A
loading control
f
n.s.
% of total ATP6V1A
60
40
20
0
IAA:
-
+
g
DMSO
IAA 2 h
ATP6V0a2 / GM130
ATP6V0a2 / LAMP2
5 µm

Supplementary Figure 4.

a) Representative confocal images of Sac1-degron A431 cells transiently transfected with GalT-RpHLuorin2 and co-stained with Golgin-97.

b) Representative pH calibration curve of Sac1-degron A431 cells transiently transfected with GalT-RpHLuorin2. Calibration buffers of pH 4.5, 5.5, 6.5 and 7.5 were used. Y-axis shows lifetime (ns), and x-axis shows the pH.

c) Representative phasor plots of FLIM imaging of Sac1-degron A431 cells transiently transfected with GALT1-RpHLuorin2 treated with DMSO or IAA for 1 and 3 h. Dotted red line shows the shift in lifetime.

d) Western blot analysis of Sac1-degron A431 cell lysates treated for 0, 1, 2 or 4 h with IAA. Stained for ATP6V_0d1 or ATP6V_1A.

e) Western blot analysis of membrane and cytosolic fractions of A431 Sac1-degron cells treated for 2 h with DMSO or IAA. Blotted for ATP6V_0d1 or ATP6V_1A.

f) Graph depicting the percentage of total ATP6V_1A subunit in the cytosol. n = 4 independent experiments. Two-tailed Wilcoxon matched-pairs signed rank test, n.s. p=0.25.

g) Representative confocal images of Sac1-degron A549 cells treated with DMSO or IAA for 2 h and stained for ATP6V_0a2, GM130 and LAMP2.

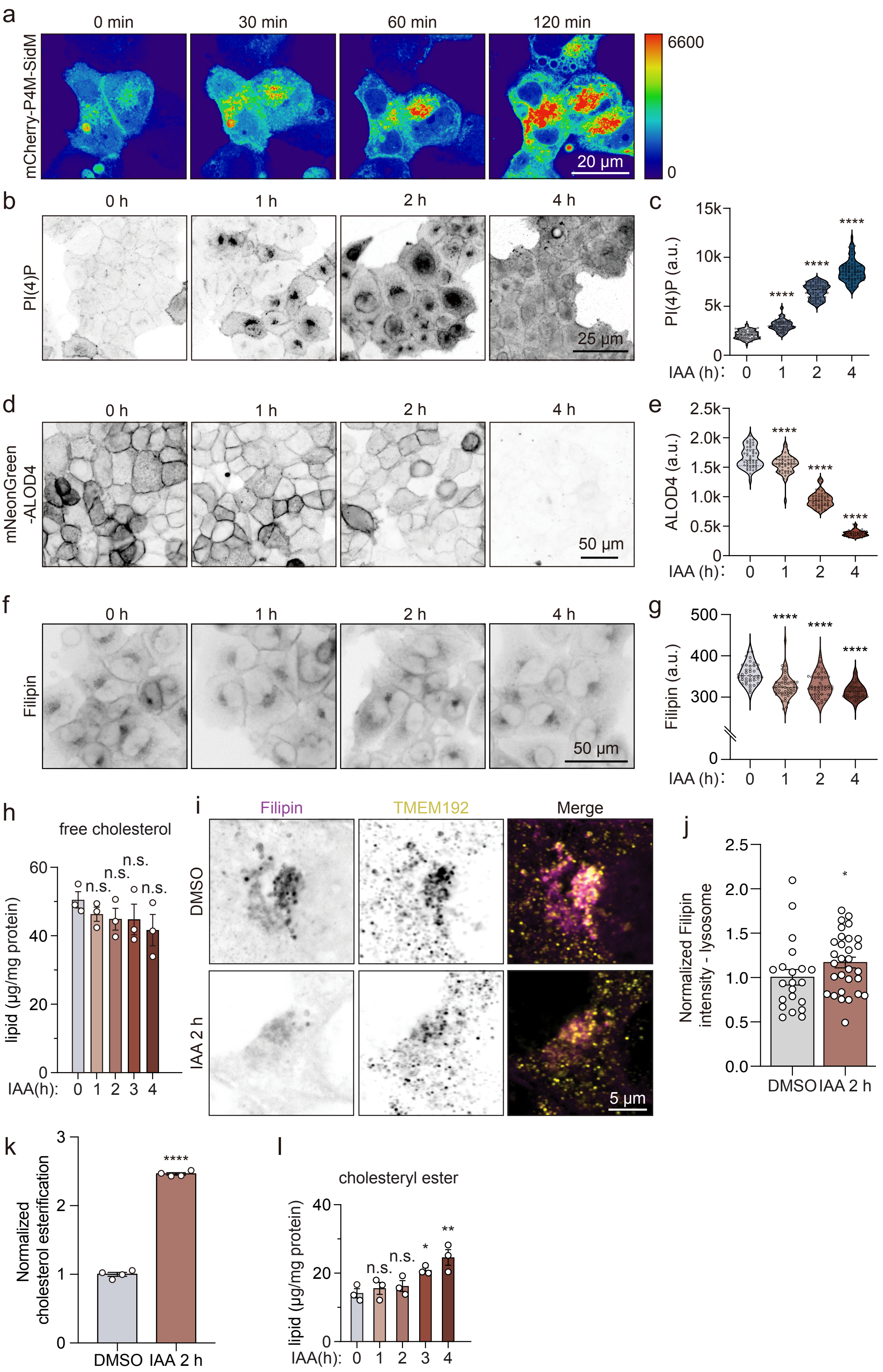
a
0 min
30 min
60 min
120 min
mCherry-P4M-SidM
6600
0
20 µm
b
0 h
1 h
2 h
4 h
PI(4)P
25 µm
c
PI(4)P (a.u.)
15k
10k
5k
0
IAA (h): 0 1 2 4
****
d
mNeonGreen
-ALOD4
50 µm
e
ALOD4 (a.u.)
2.5k
2.0k
1.5k
1.0k
0.5k
f
Filipin
50 µm
g
Filipin (a.u.)
500
400
300
h
free cholesterol
lipid (µg/mg protein)
60
40
20
IAA(h): 0 1 2 3 4
n.s.
i
Filipin
TMEM192
Merge
DMSO
IAA 2 h
5 µm
j
Normalized Filipin
intensity - lysosome
2.5
2.0
1.5
1.0
0.5
0.0
DMSO
IAA 2 h
*
k
Normalized
cholesterol esterification
3
2
1
0
DMSO IAA 2 h
****
l
cholesteryl ester
lipid (µg/mg protein)
40
20
0
IAA(h): 0 1 2 3 4
n.s.
*
**

Supplementary Figure 5.

a) Representative images of Sac1-degron A431 cells transfected with mCherry-P4M-SidM and treated with IAA for 0, 30, 60 or 120 min. mCherry-P4M-SidM intensity is converted into a heat map (0–6600 a.u.).

b) Representative widefield images of Sac1-degron A431 cells treated with DMSO for 4 h (0) or IAA for 1, 2 or 4 h and stained with PI(4)P antibody.

c) Violin plot depicting the average PI(4)P staining intensity from (**b**). Representative data from one of three independent experiments. One-way ANOVA with Dunnett's multiple comparisons test. **** $p < 0.001$.

d) Representative widefield images of Sac1-degron A431 cells treated with DMSO 4 h (0) or IAA for 1, 2 or 4 h and stained with mNeonGreen-ALOD4.

e) Violin plot depicting the average intensity of mNeonGreen-ALOD4 calculated from (**d**). Representative data from one of three independent experiments. One-way ANOVA with Dunnett's multiple comparisons test. **** $p<0.001$.

f) Representative images of Sac1-degron A431 cells stained for free cholesterol with Filipin and treated with IAA for 0 to 4 h.

g) Violin plot depicting the distribution of the average Filipin intensity calculated from (**f**). Representative data from one of three independent experiments. One-way ANOVA with Dunnett's multiple comparisons test. **** $p<0.001$.

h) Bar graph depicting the average content (µg/mg protein) of free cholesterol (FC)±S.E.M. in Sac1-degron A431 cells treated with IAA for 0 to 4 h as measured by HPTLC. n = 3 independent experiments. One-way ANOVA with Dunnett's multiple comparisons test. n.s. $p=0.8102$, $p=0.6281$, $p=0.6193$, $p=0.2804$.

i) Representative confocal images of Sac1-degron A431 treated for 2 h with DMSO or IAA and stained for Filipin and TMEM192.

j) Bar graph showing normalized Filipin intensity in the lysosomes from (**i**). n = 3 independent experiments. Two-tailed Mann-Whitney U test. * $p=0.0326$.

k) Bar graph showing the average alkyne-oleic acid incorporation into cholesteryl esters ±S.E.M. Alkyne-OA was loaded during the last 30 min of 2 h DMSO or IAA treatment in Sac1-degron A431 cells. n = 4 biological replicates. Two-tailed unpaired Student's t-test. **** $p < 0.0001$.

l) Bar graph depicting the average content (µg/mg protein) of cholesterol ester (CE) ±S.E.M. in Sac1-degron A431 cells treated with IAA for 0 to 4 h as measured by HPTLC. n = 3 independent experiments. One-way ANOVA with Dunnett's multiple comparisons test. n.s. $p = 0.9205$, $p = 0.7674$, * $p = 0.0452$, ** $p = 0.0034$.

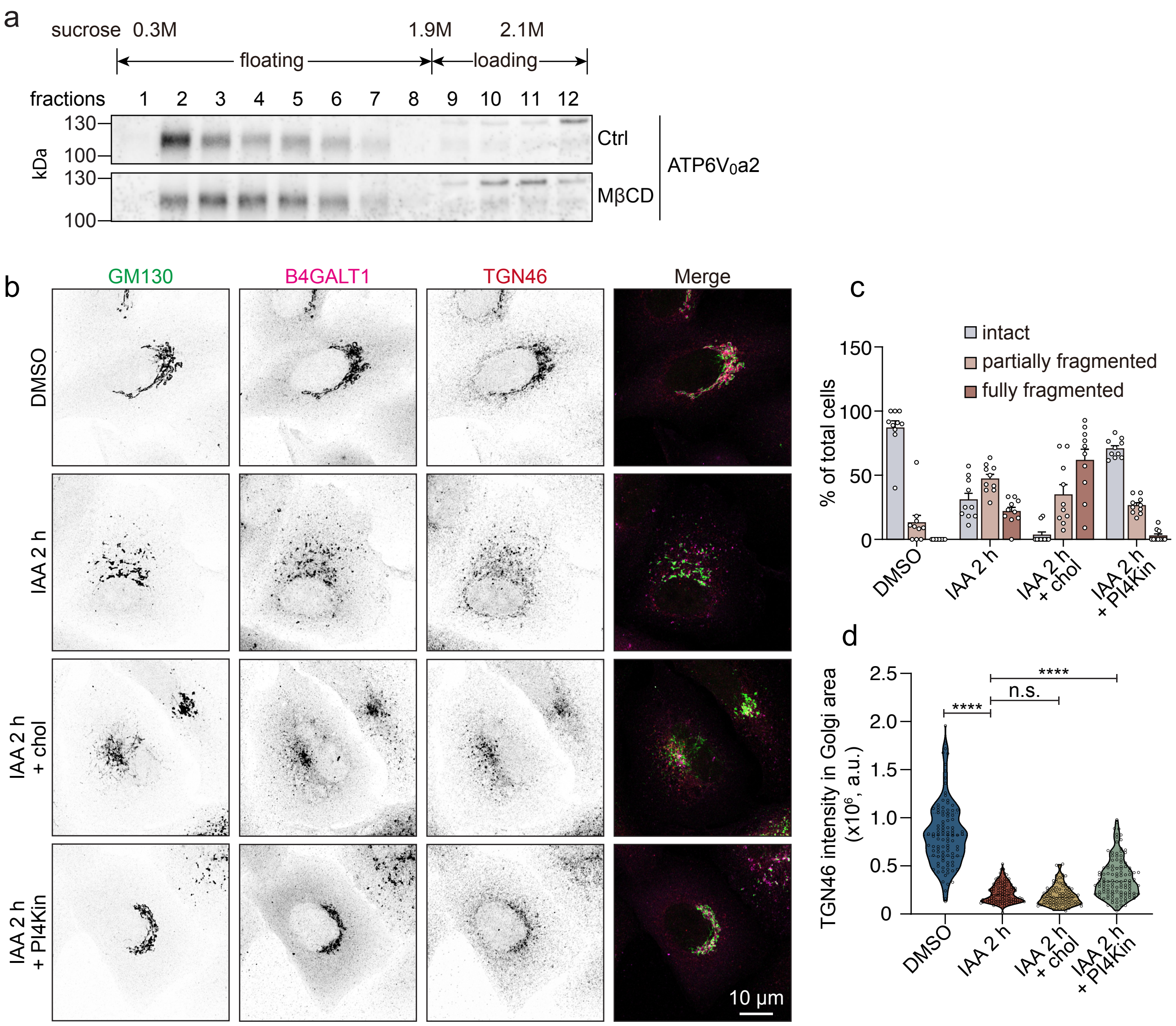

a
sucrose 0.3M
1.9M
2.1M
floating
loading
fractions 1 2 3 4 5 6 7 8 9 10 11 12
kDa
130
100
130
100
Ctrl
MβCD
ATP6V0a2
b
GM130
B4GALT1
TGN46
Merge
DMSO
IAA 2 h
IAA 2 h + chol
IAA 2 h + PI4Kin
10 µm
c
intact
partially fragmented
fully fragmented
% of total cells
150
100
50
0
DMSO
IAA 2 h
IAA 2 h + chol
IAA 2 h + PI4Kin
d
TGN46 intensity in Golgi area (x10^6, a.u.)
2.5
2.0
1.5
1.0
0.5
0
****
n.s.
****
DMSO
IAA 2 h
IAA 2 h + chol
IAA 2 h + PI4Kin

Supplementary Figure 6.

a) Western blot analysis of sucrose gradient fractionations (sucrose concentration from 0.3M (fraction 1) to 2.1M (loading fractions 9-12)) of Sac1-degron A431 cells treated with or without methyl-β cyclodextrin (MβCD) and blotted for ATP6V_0a2.

b) Representative confocal images of Sac1-degron A549 cells treated with DMSO, IAA, IAA with cholesterol or IAA with PI4K inhibitors for 2 h. Stained for GM130, B4GALT1 and TGN46.

c) Bar graph depicting the percentage (mean ±S.E.M) of cells with intact, partially fragmented, or fully fragmented Golgi in (**b**). n = 101 - 139 cells.

d) Violin plot showing the average TGN46 intensity in the Golgi area (GM130) of (**b**). n = 3 independent experiments. Kruskall-Wallis test with Dunnet's multiple correction test. n.s. $p>0.999$, **** $p<0.0001$.

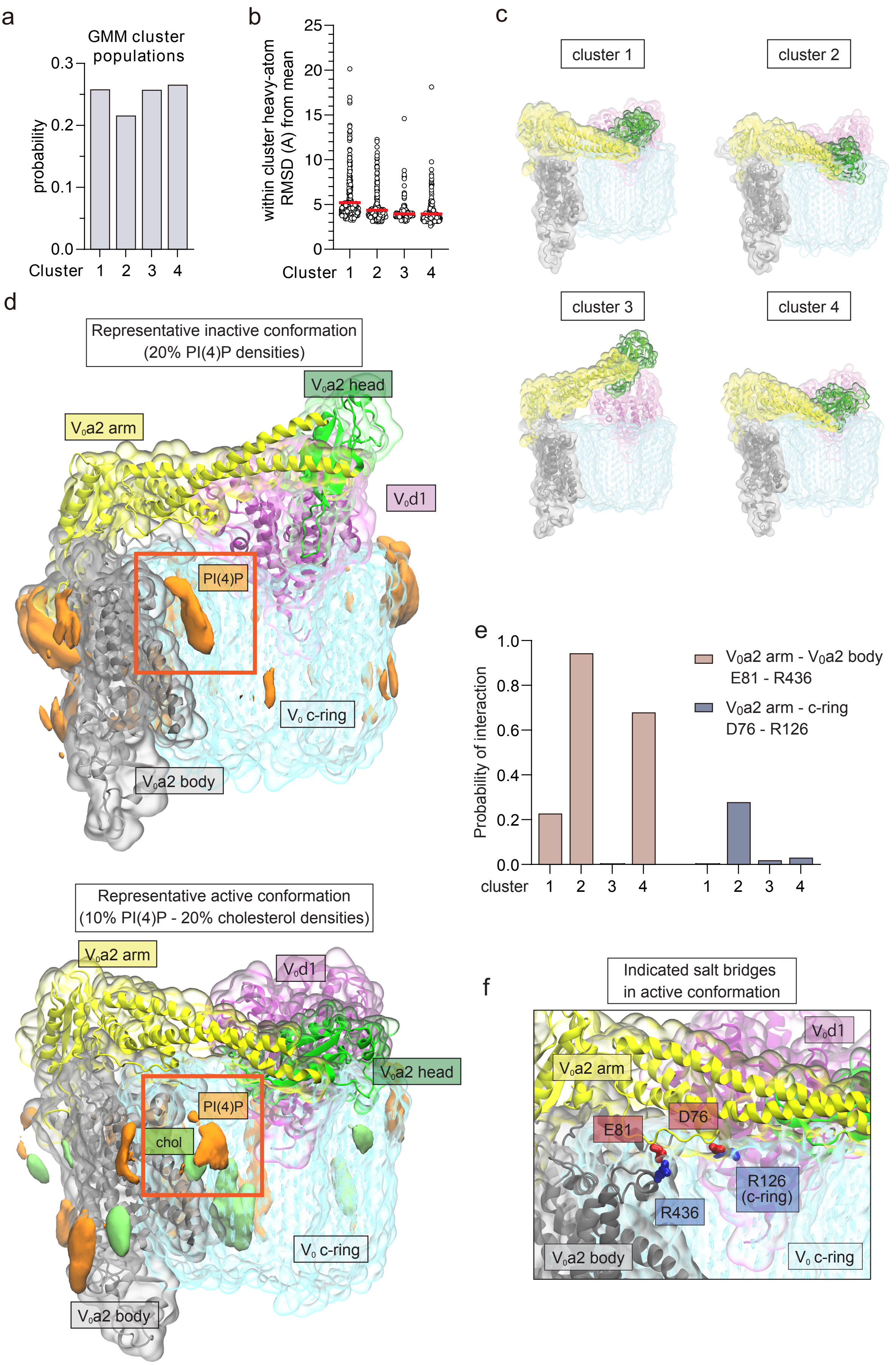
a
GMM cluster populations
probability
0.0
0.1
0.2
0.3
Cluster 1 2 3 4
b
within cluster heavy-atom RMSD (A) from mean
0
5
10
15
20
25
Cluster 1 2 3 4
c
cluster 1
cluster 2
cluster 3
cluster 4
d
Representative inactive conformation (20% PI(4)P densities)
V₀a2 head
V₀a2 arm
V₀d1
PI(4)P
V₀ c-ring
V₀a2 body
Representative active conformation (10% PI(4)P - 20% cholesterol densities)
V₀a2 arm
V₀d1
V₀a2 head
PI(4)P
chol
V₀ c-ring
V₀a2 body
e
Probability of interaction
0.0
0.2
0.4
0.6
0.8
1.0
V₀a2 arm - V₀a2 body E81 - R436
V₀a2 arm - c-ring D76 - R126
cluster 1 2 3 4 1 2 3 4
f
Indicated salt bridges in active conformation
V₀d1
V₀a2 arm
E81
D76
R126 (c-ring)
R436
V₀a2 body
V₀ c-ring

Supplementary Figure 7.

a) The four clusters identified by BGMM have similar populations indicative of balanced sampling.

b) Scatter plot (red line = mean) of Root Mean Square Deviation (RMSD) of heavy atoms of all structures in the same cluster, with the cluster mean being ~5 Å, indicative of high structural similarity within each cluster.

c) Representative side view of the V_0 region from each of the four clusters. In each cluster, it is chosen as the structure with the smallest distance from the mean of the Gaussian in the BGMM.

d) Occupational density maps of the lipids in the vicinity of the V_0 region (occupancy level 45%). Representative images of active and inactive conformations of the V_0 region showing the densities of the lipids. Upper: a representative inactive conformation (20% PI(4)P). Lower: a representative active conformation (10% PI(4)P (orange), 20% cholesterol (green)). Red rectangles show the positions of the insets presented from top in **Fig. 5j**.

e) Probability of forming the salt bridge E81 (V_0a2 arm) – R436 (V_0a2 body) and D76 (V_0a2 arm) – R126 (V_0 c-ring) in the four clusters.

f) Representative images of the V_0 region showing the localization of the two salt bridges: E81 (V_0a2 arm) – R436 (V_0a2 body) and D76 (V_0a2 arm) – R126 (V_0 c-ring). Of note, the two salt bridges cannot form simultaneously, therefore the representative active conformation presents a conformation where the involved residues are closest together.

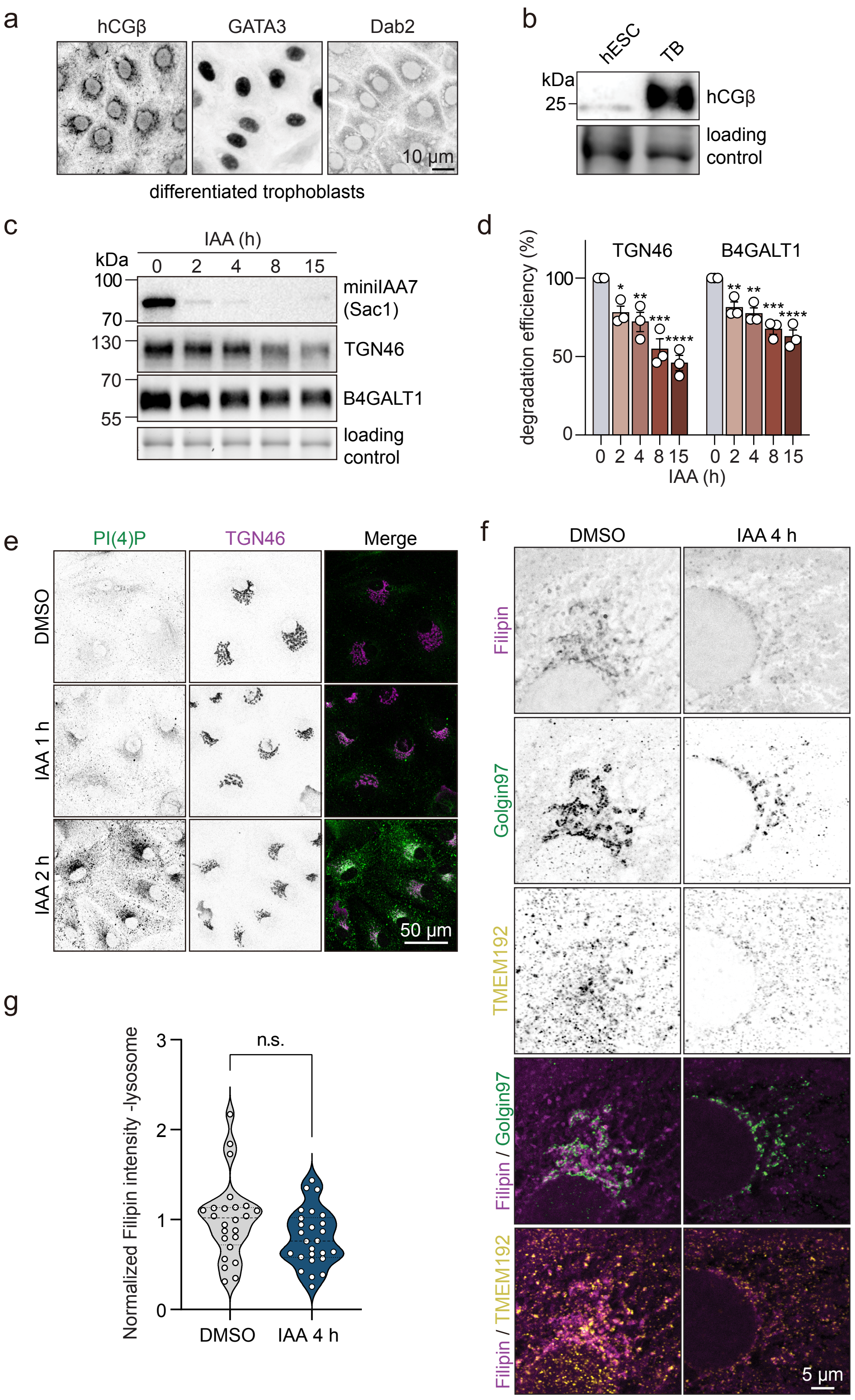
a
hCGβ
GATA3
Dab2
10 µm
differentiated trophoblasts
b
hESC
TB
kDa
25
hCGβ
loading control
c
IAA (h)
kDa
0 2 4 8 15
100
70
miniIAA7 (Sac1)
130
TGN46
70
55
B4GALT1
loading control
d
TGN46
B4GALT1
degradation efficiency (%)
100
50
0
*
**
***
****
0 2 4 8 15 0 2 4 8 15
IAA (h)
e
PI(4)P
TGN46
Merge
DMSO
IAA 1 h
IAA 2 h
50 µm
f
DMSO
IAA 4 h
Filipin
Golgin97
TMEM192
Filipin / Golgin97
Filipin / TMEM192
5 µm
g
Normalized Filipin intensity -lysosome
n.s.
3
2
1
0
DMSO
IAA 4 h

Supplementary Figure 8.

a) Representative fluorescence micrographs of wild type hESCs differentiated into trophoblasts and stained for the trophoblast markers hCGβ, GATA3 and Dab2.

b) Western blot analysis of hESC and TB lysates blotted for hCGβ.

c) Western blot analysis of Sac1-degron STB treated for 0, 2, 4, 8 or 15 h with IAA. Blotted for miniIAA7 (Sac1), TGN46 and B4GALT1.

d) Bar graph depicting the TGN46 or B4GALT1 degradation efficiency calculated from **(c)**. n = 3 biological replicates. One-way ANOVA with Dunnett's multiple comparisons test. TGN46: * p=0.033, ** p=0.0087, *** p=0.0003, **** p<0.0001, B4GALT1: ** p=0.0084, p=0.0025, *** p=0.0002, **** p<0.0001.

e) Representative confocal images of Sac1-degron TBs treated with DMSO or IAA for 1 to 2 h and stained for PI(4)P and TGN46.

f) Representative confocal images of Sac1-degron TBs treated for 4 h with DMSO or IAA and stained for Golgin-97, TMEM192 and filipin.

g) Violin plot showing normalized Filipin intensity in the lysosomes of (**f**) n = 3 independent experiments. Two-tailed Mann-Whitney U test. n.s. p=0.0894.
