# Supplementary material for: Control of Golgi- V-ATPase through Sac1-dependent co-regulation of PI(4)P and cholesterol
Source: Nat Commun. 2025 Aug 21;16:7808. doi: 10.1038/s41467-025-63125-7 (PMC12371084; doi:10.1038/s41467-025-63125-7)
Supplement: Supplementary file 2 — Description of Additional Supplementary Files [file 41467_2025_63125_MOESM2_ESM.pdf]

Description of Additional Supplementary Files

File Name: Supplementary Data 1

Description: Quantification of Golgi morphological changes from Transmission Electron Microscopy images of Sac1-degron A431 cells treated with DMSO or IAA for 2 h.

File Name: Supplementary Data 2

Description: Results of the proteomics analysis of Sac1-degron A431 cells treated with DMSO or IAA for 3 h. The first tab contains a summary of the MS analysis, showing data normalization, coefficients of variation and sample correlation. The other tabs contain the differential abundance of the proteins, the detected proteins, list of downregulated and upregulated proteins, and the respective GO analysis of the down- and upregulated proteins from the Zenodo GO database.

File Name: Supplementary Movie 1

Description: Partial Least Squares based Discriminant Analysis (PLS-DA) of the four clusters. Movie highlighting the largest structural changes of the V_0 region from top view as it moves from clusters 1, 3 and 4 (impairing assembly) to cluster 2 (favoring assembly).

File Name: Supplementary Movie 2

Description: Partial Least Squares based Discriminant Analysis (PLS-DA) of the four clusters. Movie highlighting the largest structural changes of the V_0 region from side view as it moves from clusters 1, 3 and 4 (impairing assembly) to cluster 2 (favoring assembly).
